# Higher Incidence and Longer Recovery Time from Non-Contact Muscle Injuries in *ACTN3* XX Genotype Players from a Soccer Academy: A Three-Season Longitudinal Study

**DOI:** 10.1186/s40798-026-00978-2

**Published:** 2026-01-31

**Authors:** Benjamin Barthelemy, Guillaume Ravé, Juan Del Coso, Ayoub Saeidi, El Mokhtar El Ouali, Benoit Bideau, Urs Granacher, Hassane Zouhal

**Affiliations:** 1 Movement, Sport, Health and Sciences laboratory (M2S). UFR-STAPS, University of Rennes 2-ENS Cachan, Av. Charles Tillon, 35044 Rennes Cedex, France; 2 Toulouse Football Club, 31000 Toulouse, France; 3 Sport Sciences Research Centre, Rey Juan Carlos University, Fuenlabrada, Spain; 4 Department of Physical Education and Sport Sciences, Faculty of Humanities and Social Sciences, University of Kurdistan, Sanandaj, Kurdistan Iran; 5 Department of Biology, Laboratory of Biology and Health, Ibn Tofail University of Kenitra, Kenitra, Morocco; 6 Department of Sport and Sport Science, Exercise and Human Movement Science, University of Freiburg, Freiburg, Germany; 7 International Institut of Sport Sciences (2IS), 35000 Rennes, France

**Keywords:** Football, SNP, Genetic, Team sport, Injury epidemiology, High-performance

## Abstract

**Objective:**

The purpose of this study was to examine whether the *ACTN3* R577X polymorphism was associated with injury rate and recovery time from non-contact muscle injuries in youth academy players and professional soccer players.

**Methods:**

The *ACTN3* rs1815739 genotype was identified in 76 male soccer players (22 professional, 27 U19 and 27 U17) from a top-level French soccer club. Over three consecutive competitive seasons (2020/21 to 2022/23), the players were prospectively monitored. The club’s medical staff systematically recorded all injuries sustained during soccer exposure. Injury incidence was calculated based on total soccer exposure, and return-to-play time (RTT) for each injury was determined by the medical staff. A total of 312 injuries were documented, including 144 non-contact muscle injuries. Injury incidence rates (IRs) and rate ratios (RRs) were compared across player genotypes, both overall and within each category, using Poisson or negative binomial regression models with exposure time as an offset. RTT was analyzed by genotype using the Kruskal–Wallis test.

**Results:**

Overall genotype distribution was RR, 52.6%; RX, 30.3%; and XX, 17.1%. Across all players, XX carriers had the highest injury incidence (8.54 [6.54–10.39]/1000 h) followed by RX players (6.65 [5.39–7.91]/1000 h) and RR players (5.15 [4.35–5.95]/1000 h), although these differences did not reach statistical significance. The RRs for XX compared with RR players was 1.66 (95% CI: 0.85–3.23, *p* = 0.140), indicating a non-significant tendency toward higher incidence in XX players. However, RTT differed significantly among genotypes (*p* = 0.007), with median [IQR] values of 13 [10, 16] days for RR, 16 [14, 22] days for RX, and 18 [13, 19] days for XX. Subgroup analyses showed that RTT differences were significant in U17 players (*p* = 0.004), with XX requiring longer recovery (23 days) compared to RR players (11 days). However, these genotype-related differences in RTT were not significant among professional soccer players.

**Conclusion:**

The *ACTN3* R577X polymorphism was associated with recovery characteristics following non-contact muscle injuries in soccer players. Specifically, players with the XX genotype required significantly longer return-to-play times, a pattern evident in youth academy players but not in the professional group.

## Key Points

- The distribution of the *ACTN3* XX genotype was comparable between professional soccer players and those in youth academy categories (U17 and U19).
- When pooling all players, return-to-play time after a non-contact muscle injury was significantly longer in *ACTN3* XX compared with RR players, with RX showing intermediate values.
- Subgroup analyses revealed that this association with longer return-to-play duration was most pronounced among youth academy players, while no statistically significant differences were observed in the professional group.

## Introduction

Soccer (football) is a high-intensity, contact-based team sport that requires players to repeatedly perform explosive actions, including rapid accelerations and decelerations, sprinting, changes of direction, as well as frequent jumping and landing tasks [1]. Therefore, contact and non-contact injuries are relatively common phenomenon for professional soccer (football) players [2]. In professional football, injury incidence is around 36 injuries/1000 hours of exposure during matches and 3.7 injuries/1000 hours of exposure during training activities [3]. Injury incidence in young soccer players is slightly lower with 14.8 injuries/1000 hours of game exposure and 2.3 injuries/1000 hours of training exposure [4]. Muscle injuries represent about one third of the total injuries with about 0.4 muscle injuries per match [5]. Interestingly, the incidence of muscle injuries has been increasing at a rate of around 2% per year and hamstring injuries in men’s professional football have risen by 4% annually since 2001 [6]. This trend likely reflects the growing physical demands of the sport, as well as the continued need for more effective muscle injury prevention strategies.

Overall, return-to-play after an injury depends on the type of injury but it can go from 1 to 2 weeks for mild muscle injuries to 6–12 months for knee ligament injuries [7] The impact of such injuries extends beyond its sporting consequences, as they can adversely affect the player’s overall health. Additionally, injuries is a major problem for soccer coaches [8] because a high incidence of injury is negatively related to worse final ranking in the championship [9] and for clubs because implies indirect losses from player absences, decreased team performance, and potential reductions in sponsorships or prize money [10]. Intrinsic factors, such as a player’s age, genetics, previous injury history, and muscle strength imbalances, are inherent to the individual and may predispose them to injury. Extrinsic factors, such as acute and cumulative training and match load, playing surface, footwear, and level of play, are related to the environment and external demands placed on the athlete [11]. Among the various potential contributing factors, genetics is increasingly recognized as an intrinsic risk factor for soccer injuries, particularly for those with a non-contact mechanism. Specific genetic variations may predispose athletes to conditions such as muscle strains, ligament tears, and tendinopathies [52].

Recently, many studies have focused on the relationship between the human genome and physical function [12] and some gene polymorphisms might be involved in non-contact muscle injuries [13–15]. As a result, genetic testing in athletes is increasingly being used for injury prevention [16, 17]. Genetics plays a significant role in determining an individual’s response to their environment, including athletic performance and susceptibility to musculoskeletal injuries [18]. The risk of injury and recovery from injury can be influenced by the expression of certain proteins, which can have different features depending on genetic variants in the genes that encode them. Among the most common genetic variants are single nucleotide polymorphisms (SNPs), defined as single base-pair changes in the DNA sequence that occur in more than 1% of the population [53]. SNPs represent the most frequent type of genetic variation and can be found in both coding (gene) and non-coding regions of the genome. Although a SNP is a variation at a single position in the DNA sequence, it can affect how genes are expressed, and influence an individual’s susceptibility to diseases or to injury. Several SNPs in genes coding key proteins for muscle performance and reparation has been identified as with potential influence on risk and recovery from sporting injuries, particularly the p.R577X in the α-actinin-3 (*ACTN3)* gene [13, 21].

*ACTN3* is one strong evidence of the relation between genotype, athletic characteristic and injuries in sport [13, 20, 21]. This gene encodes the protein α-actinin-3 which is a structural component of the Z-disc in skeletal muscle [22]. Expression of α-actinin-3 is limited to type II muscle fibers [22] and for this reason, this protein is considered key to product explosive strength and fast, powerful muscle contractions [23, 24]. In the last few decades, there has been a high scientific interest about the phenotypical consequences of the stop codon variant p.R577X in the *ACTN3* gene [25]. Usually known as the R577X polymorphism, this genetic change leads to the replacement of an arginine (R) with a premature stop codon (X) at amino acid 577 [21]. Individuals homozygous for this stop codon variant (XX genotype) are characterized by lack of expression of α-actinin-3 in muscles, opposed to the RR or RX polymorphism that express functional α-actinin-3 [25, 26]. XX genotype is estimated that ~ 20% of the world population [20] and it is not linked to any serious disease but has been linked with several deleterious effect in humans, particularly athletes.

A few studies have found that the *ACTN3* XX genotype predispose athletes to a higher probability of some non-contact injuries compared to RR athletes, including soccer players [13, 27] Additionally, XX athletes present higher levels of exercise-induced muscle damage [28–30] and has been linked to lower muscle strength, reduced muscle volume, and impaired capacity for explosive muscular actions when compared to athletes of the same level but with the RR genotype [13]. Last, the XX genotype may have might be associated not only with a higher risk of non-contact muscle injuries, but also of recovery time from these conditions [31].

The investigations mentioned above have detailed the negative effect of the XX genotype on the risk of injury and recovery time of non-contact muscle injuries of adult and elite athletes. One study conducted in youth academy soccer players [50] reported an association between the *ACTN3* X allele and longer return-to-play times following ankle injuries. However, to our knowledge, no studies have specifically investigated whether the increased injury risk linked to the XX genotype is also evident in younger players when focusing exclusively on non-contact muscle injuries. For this reason, we aimed to analyze the association of *ACTN3* R577X polymorphism with the rate of, and recovery time from non-contact muscle injuries in a soccer club that possess different categories, from a professional team to academy teams. We hypothesized that players with the XX genotype would have a higher risk of non-contact muscle injury and longer times to return to play than RR players and this effect would be present in all categories analyzed.

## Methods

### Experimental Design

**Fig. 1:**
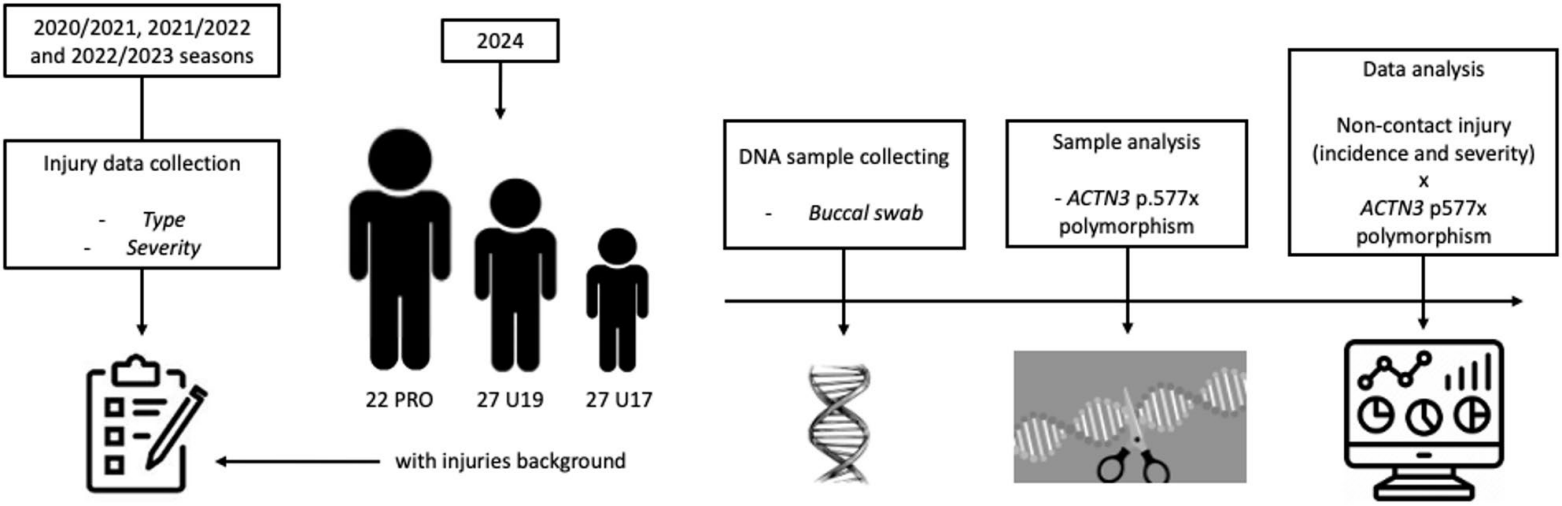
Experimental protocol diagram

In this prospective observational cohort study, we aimed to determine the association, if any, of the *ACTN3* genotype (RR vs. RX vs. XX) with injury incidence and time to return to play from soccer injuries in professional and young soccer players. The protocol is presented in Fig. 1 and the details of injury data collection are outlined in the section *Injury data collection.*

### Participants

**Fig. 2:**
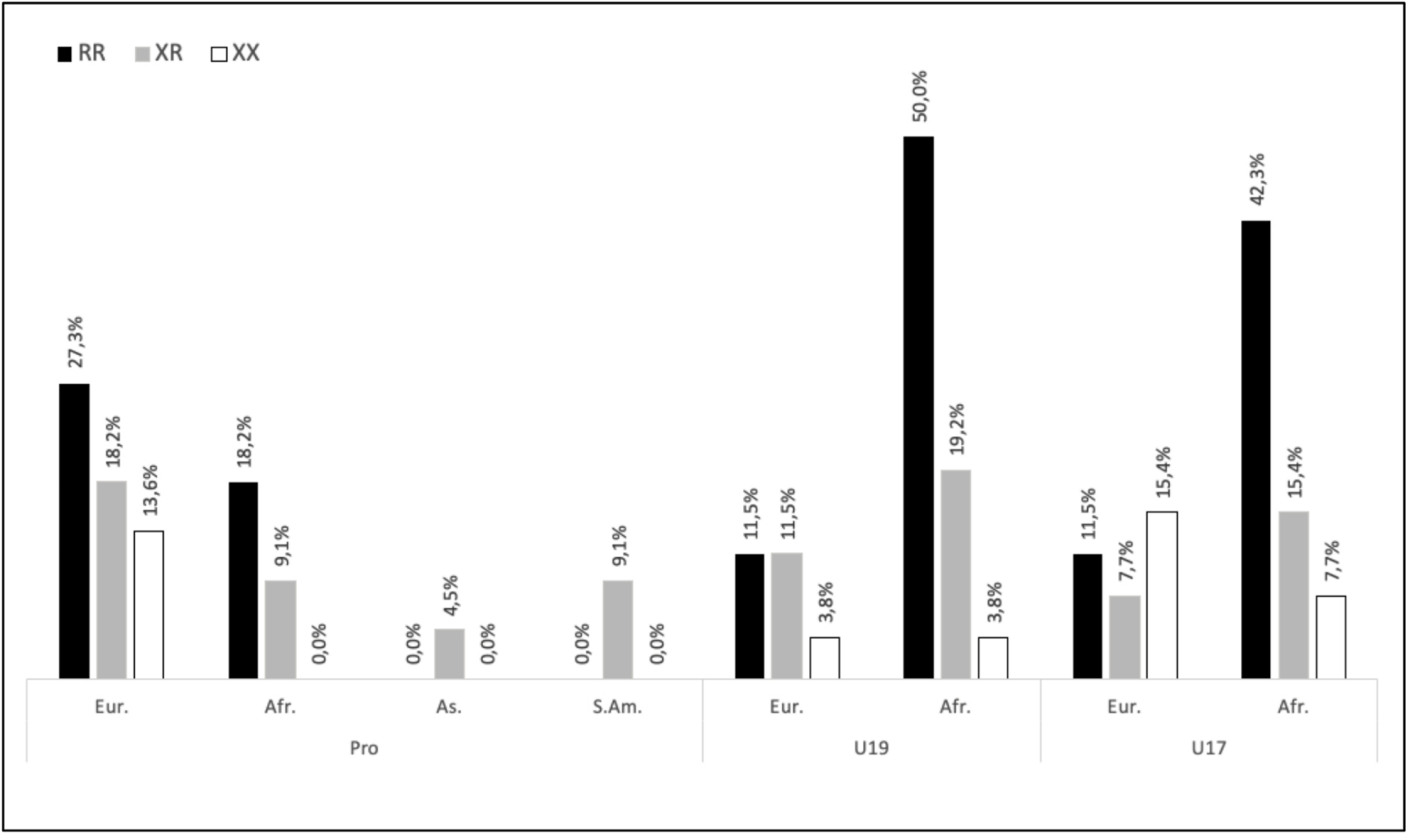
Frequency of genotypic distribution based on the ethnic origin of the players. (Eur: European, Afr: African, As: Asian, S.Am: South American)

Initially, 77 soccer players volunteered to participate in the study. The success rate of genetic profiling was 98.7% as the ACTN3 genotype of one U19 player was not clearly identified in the genotyping analysis. Therefore, this participant was excluded from genotype-based analyses, resulting in a final sample of 76 players. Table 1 shows the age and anthropometric characteristics of the final sample (22 professional, 27 U19, and 27 U17 players). The ethnic origin of participants is illustrated in Fig. 2.

**Table 1:** Main anthropometric characteristics, field position and ethics origin of the players

	PRO	U19	U17
Number, n (%)	22 (28.9)	27 (35.5)	27 (35.5)
Age (years)	24.3 ± 3.2	18.8 ± 0.8	16.6 ± 0.5
Body mass (kg)	79.0 ± 5.7	70.0 ± 6.6	67.9 ± 7.1
Body fat (%)	8.4 ± 1.5	9.0 ± 1.6	8.8 ± 1.6
Defenders, n (%)	9 (40.9)	9 (33.3)	10 (37.0)
Midfielders, n (%)	6 (27.3)	7 (25.9)	7 (25.9)
Forwards, n (%)	7 (31.8)	11 (40.7)	10 (37.0)
European	13 (59.1)	7 (26.9)	9 (34.6)
African	6 (27.2)	19 (73.1)	17 (65.4)
Asian	1 (4.5)	0 (0)	0 (0)
South American	2 (9.1)	0 (0)	0 (0)

Data are numbers (and frequencies in percentage) or mean ± standard deviation (SD). PRO = Professional football players

All players were from the same club based in France, and the professional players played in the French Ligue 1, and the U19 and U17 players played at the highest national level in their category. So, independently of their category, all teams were playing at their maximal level and can be considered high-performance athletes. Players trained approximately 5 times a week and played 1 game per week during the seasons. As all players were in the same club, they shared the same characteristics for the management of load, participated in comparable injury prevention programs and had access to the same treatments for injury recovery.

### Sample Collection and Genotyping

A genomic DNA sample was obtained via buccal swab with a cotton swab (EUROTUBE^®^ Collection Swabs, Deltalab, Spain), performed by the club’s medical staff. Afterwards, anthropometric variables (i.e., body mass, height and body fat percentage using the 4-skinfold method [33]), were measured. The DNA samples were identified with an alphanumeric code to avoid identifying the participants, and the samples were sent to a certified genetics laboratory for genotyping. The laboratory provided genotype identification for each alphanumeric code/sample, and the principal investigator was then responsible for linking the laboratory information with the players’ data to prevent the identification of players’ identities during the genotyping process. If genotype assignment failed, the participant was excluded from the analysis. Only the principal investigator has access to the link between the alphanumeric code and the player’s name.

To avoid contamination, recommendations for molecular genetics laboratories were followed, including physically isolated work area laboratories for each process (sample manipulation and extraction). In addition, reference samples (internal controls, blank samples, and negative controls) and contamination monitoring in all steps were included. Specifically, genomic DNA was isolated using an organic-based DNA extraction method adapted to Amicon^®^ Ultra-0.5 mL columns (Sigma-Aldrich, Madrid, Spain), including a elution of the sample in 50 µL of interval control to obtain a solution with a DNA concentration of ~ 30 ng/mL. Positive controls for all genotypes were used from the Mexican branch of CANDELA Consortium [20]. Genotyping of ACTN3 rs1815739 polymorphism (c.1858 C > T; p.R577X) was conducted using a TaqMan SNP Genotyping Assay (Assay ID: C___590093_1_; Applied Biosystems) and the reaction was performed in an Applied Biosystems 7500 Fast Real-Time PCR System (Applied Biosystems, Foster City, CA, USA). Each genotyping reaction had a final volume of 10 µL, consisting of 5 µL of TaqMan^®^ Genotyping Master Mix (Applied Biosystems), 0.5 µL of the specific TaqMan^®^ SNP Genotyping Assay, 2.5 µL of nuclease-free water (Thermo Fisher Scientific, Waltham, MA, USA), and 2 µL of genomic DNA (~ 30 ng/mL). The results were analyzed using 7500 Software v2.0.5 (Applied Biosystems). DNA analyses that did not report a clear ACTN3 genotype were repeated. From the total, 60 samples were randomly selected, and they were genotyped a second time. We confirmed that the genotyping results perfectly agreed between duplicates which reinforces the accuracy of the analysis.

### Injury Data Collection

The questionnaire used to collect the injuries was based on the consensus statement on injury definitions and data collection in epidemiological studies of the International Olympic Committee (IOC) [32]. Throughout the three-season period, all injuries were systematically recorded by each team’s medical staff, including detailed information on injury type, anatomical location, mechanism (contact vs. non-contact), and severity (Fig. 1). For the purpose of this study, only non-contact muscle injuries affecting the lower limbs were extracted from the full dataset and included in the analysis. Muscle injuries were diagnosed on the basis of a clinical examination by the team’s medical staff, and when deemed necessary, confirmed and morphologically classified using ultrasound and/or magnetic resonance imaging (MRI). For the recording of injuries, the medical staff used an ad hoc spreadsheet (date of injury, location on the body, type of injury, with or without contact, date of return to train) created by the research group of this study. The spreadsheet was completed either on the day of the injury or within 24 hours of its occurrence. The follow-up of each injury case was concluded once the player returned to play. Traumatic muscle injuries caused by contact with another player or produced during tackle actions were discarded from the analysis because they are potentially unaffected by the player’s genotype. To standardize the incidence of injuries among genotypes, the incidence of injuries was calculated per player as the number of injuries per 1000 h of football exposure [3]. Importantly, exposure time to training and matches was accurately documented, as this data is routinely collected by teams on an individual basis for each player. This allowed for precise calculation of injury incidence rates, taking into account both the number of injuries and the actual exposure time. Players could sustain anywhere from zero to multiple injuries per season. All injuries meeting the inclusion criteria were recorded and used to calculate injury incidence. The time to return to train was calculated as the time (in days) elapsed between the injury and the moment when the player is deemed ‘fit’ by the medical team to resume full team training, not the return to competition, in order to avoid bias from potential coaching decisions that might delay the return to play for technical or tactical reasons rather than the player’s availability.

### Statistical Analyses

We determined whether the *ACTN3* genotype distribution in the sample met the Hardy–Weinberg Equilibrium (HWE) using a Chi-square test (χ² test). A χ² test was also used to verify if genotype frequencies were similar across teams. For categorical variables (e.g., field position, category, and genotype), descriptive statistics were calculated as frequencies for each genotype. Differences among genotypes for categorical variables were assessed using χ² tests; in the case of significance, standardized residuals were calculated to identify genotypes with an observed distribution differing from the expected distribution.

For injury incidence, which is based on count data (number of injuries relative to exposure), we used Poisson regression models with exposure (in hours) included as an offset. In cases of overdispersion, negative binomial regression was applied. Results are presented as incidence rates with 95% confidence intervals (CIs), and rate ratios (RRs) with 95% CIs were calculated for genotype comparisons.

For return-to-play time (days), due to the non-normal distribution of the data, differences among genotypes were analyzed using the Kruskal–Wallis test. Pairwise comparisons were performed with Dunn’s test and Bonferroni correction.

The level of significance was set at *p* < 0.05. All statistical analyses were performed using R (version 4.4.0).

### Ethics and Confidentiality

The protocol was approved by an independent French ethics committee (Comité de Protection des Personnes SUD-MEDITERRANEE III, RIPH2G, File number: 24.02439.000431) and the study was conducted in accordance with the ethical standards outlined in the Declaration of Helsinki. A researcher explained the aim of the investigation, its benefits, and risks, and written informed consent was obtained from all participants before the onset of the experiment. Participants’ rights and confidentiality were protected during the whole experiment, and the genetic information was used only for the purposes included in this investigation. In this study involving underage football players, informed consent was obtained from their parents or legal guardians prior to participation, ensuring compliance with ethical standards for research involving minors.

## Results

### Sociodemographic Variables

The number and position of the players according to their genotype is presented in Table 2. Figure 2 presents the frequency of genotypic distribution based on the ethnic origin of the players. We tested whether the distribution of player positions (defenders, midfielders, and forwards) differed across genotypes (RR, RX, XX) (Table 2). The χ² test showed no significant differences (*p* = 0.554 for PRO players; 0.854 for U19 players and 0.725 for U17 players), indicating that playing position was similarly distributed among genotypes.

**Table 2:** *ACTN3* genotype distribution according to field positions, category, and genotype

	Category	RR	RX	XX	*P* value
Number, n (%)	ALL	40 (52.6)	23 (30.3)	13 (17.1)	
	PRO	10 (45.5)	9 (40.9)	3 (13.6)	0.434
	U19	16 (59.3)	8 (29.6)	3 (11.1)	
	U17	14 (52.6)	6 (22.2)	7 (25.9)	
Defenders, n (%)	PRO	4 (18.2)	4 (18.2)	1 (4.5)	0.554
Midfielders, n (%)		2 (9.1)	3 (13.6)	1 (4.5)	
Forwards, n (%)		4 (18.2)	2 (9.1)	1 (4.5)	
Defenders, n (%)	U19	4 (14.8)	4 (14.8)	2 (7.4)	0.854
Midfielders, n (%)		4 (14.8)	2 (7.4)	1 (3.7)	
Forwards, n (%)		8 (29.6)	2 (7.4)	0 (0)	
Defenders, n (%)	U17	5 (18.5)	2 (7.4)	0 (3.7)	0.725
Midfielders, n (%)		6 (22.2)	0 (0.0)	4 (14.8)	
Forwards, n (%)		3 (11.1)	4 (14.8)	2 (7.4)	

Data are numbers and frequencies (in percentage) for each genotype per category and field positions. P value: difference in the distribution of genotypes based on categories and the different field positions within each category with χ^2^ test

The genotype analyses reported the following distribution: RR, 52.6%; RX, 30.3%; and XX, 17.1%. When considering the overall sample, genotype distribution deviated from Hardy–Weinberg Equilibrium (HWE, *p* = 0.007). However, within subgroups, HWE was respected in professional players (*p* = 0.674) and U19 players (*p* = 0.234), but not in U17 players (*p* = 0.006). Genotype frequencies did not differ significantly between categories (*p* = 0.432), indicating a similar distribution pattern across professional and academy players.

### Injury Incidence

During the collection period, a total of 312 injuries were recorded, including 144 non-contact muscle injuries. Table 3 presents the incidence rates of non-contact muscle injuries (per 1000 h of exposure, with 95% CIs) and return-to-play times (median and IQR) across genotypes and categories.

**Table 3:** Incidence rates of non-contact muscle injuries per 1000 h of exposure across ACTN3 genotypes and player categories (Professional, U17, U19, and pooled groups)

Category	Genotype	Incidence (/1000 h) [95% CI]	Rate Ratio vs. RR [95% CI]	Return-to-Play (days), median [IQR]	Kruskal-Wallis (RTT)
All players	RR	5.15 [4.35–5.95]	Ref.	13 [10.0-16.2]	*p* = 0.007
	RX	6.65 [5.39–7.91]	1.29 [0.74–2.25] *p* = 0.370	16 [14.0-21.5]	
	XX	8.54 [6.54–10.39]	1.66 [0.85–3.23] *p* = 0.140	18 [13.0–19]	
Professionals	RR	5.30 [3.60-7.00]	Ref.	15 [14.0–16.0]	*p* = 0.510
	RX	6.22 [4.67–7.89]	1.17 [0.81–1.71] *p* = 0.400	18 [12.0–22.0]	
	XX	7.00 [6.00–8.00]	1.32 [0.80–2.19] *p* = 0.280	19 [16.0-23.5]	
U17	RR	5.29 [4.21–6.43]	Ref.	11 [9.0-16.2]	*p* = 0.004
	RX	5.67 [2.17–9.34]	1.07 [0.38–3.03] *p* = 0.900	23 [17.2–25.8]	
	XX	10.43 [8.57–12.71]	1.97 [0.75–5.17] *p* = 0.170	18 [18.0–24.0]	
U19	RR	4.94 [3.56–6.31]	Ref.	13 [10.8–15.8]	*p* = 0.090
	RX	7.88 [6.62–9.12]	1.59 [0.64–3.97] *p* = 0.320	15 [13.8–15.5]	
	XX	5.67 [0.00–9.00]	1.15 [0.30–4.38] *p* = 0.840	12 [7.5–12.5]	

Values are expressed as incidence rates with 95% confidence intervals (CI). Rate ratios (RR) with 95% CI were calculated for genotype comparisons using Poisson or negative binomial regression models with exposure included as an offset

Using Poisson regression models, no statistically significant differences in incidence rates were observed between genotypes when analyzing all players together (PRO + U17 + U19), nor within professional or youth subgroups. Incidence rates tended to be higher in XX players compared with RR players, particularly among U17 players (XX = 10.4 injuries/1000 h, RR = 5.3 injuries/1000 h; rate ratio = 1.97, 95% CI: 0.75–5.17, *p* = 0.17).

In contrast, return-to-play time (RTT) showed statistically significant differences between genotypes. The Kruskal–Wallis test revealed longer RTT in XX compared to RR players when considering all players together (*p* = 0.007) and specifically within the U17 subgroup (*p* = 0.004). Pairwise Dunn’s tests confirmed that XX players had longer absence times than RR players in these groups.

## Discussion

Our results suggest that the *ACTN3* XX genotype may be associated with prolonged recovery following non-contact muscle injuries in football players. Specifically, return-to-play times were significantly longer in XX compared to RR genotype players, with the most pronounced differences observed in U17 athletes. These results extend previous evidence from professional cohorts to younger populations, underscoring the potential influence of α-actinin-3 deficiency not only on injury susceptibility but also on recovery dynamics. Additionally, although the difference did not reach statistical significance, there was a noticeable trend toward a higher incidence of non-contact muscle injuries in players with the *ACTN3* XX genotype. While this finding should be interpreted with caution, it may still hold clinical relevance and warrants further investigation. These findings contribute to the existing literature and are consistent with previous studies on professional footballers, as association of the XX genotype with a higher risk of muscle injuries in football [34–36] and longer recovery times [31] has been previously reported. However, this study offers a novel contribution by demonstrating, for the first time, that this association is also evident in younger cohorts. Interestingly, a previous study involving a similar group of youth soccer players reported that the X allele was associated with prolonged recovery following ankle injuries [50]. Together, these findings suggest that the *ACTN3* R577X genotype may influence recovery outcomes across different types of injuries, even in young athletes, highlighting its potential relevance for injury management and prevention strategies in youth football. From a practical standpoint, genotype analysis of the p.R577X (rs1815739) polymorphism in the *ACTN3* gene could potentially help identify players with the XX genotype, who may require longer recovery periods following certain injuries. While these findings are promising, they should be considered preliminary and require validation in larger, independent cohorts. Nonetheless, the observed trends suggest that genetic screening may offer valuable insights for individualized injury management and rehabilitation planning, even in youth football populations. As the measurement of genetic variations is becoming less expensive per year, it seems a reasonable investment as the identification of players more prone to injury may impact on players’ health, team’s performance but also in club’s finances.

Previous research has highlighted several non-modifiable risk factors for injuries in football and other high-intensity sports, such as age, history of similar injuries, and muscle architecture [10, 37, 38]. Our findings build on existing knowledge by suggesting that genetic predisposition (specifically the *ACTN3* XX genotype) may influence not only injury susceptibility but also the recovery process following football-specific muscle injuries. Although preliminary, our findings and those of previous researchers indicate a potential association between the XX genotype or the X allele and an increased risk of muscle injuries, as well as a prolonged return-to-play time following an injury, particularly among young players [34–36]. However, not all studies have supported this association; for example, some authors have found no link between *ACTN3* genotype and hamstring injury risk in professional footballers [51]. Despite the smaller sample size of the current study, the injury recording over three competitive seasons provides novel insight into how *ACTN3* genotype may impact injury incidence and recovery in this context. This is a very important result because player availability is a crucial factor for championship outcomes [8] and for the careers of young players aiming to secure a professional contract. Additionally, since genetics is a non-modifiable factor, it is conceivable that, in the future, prevention strategies for XX players could focus on the management of modifiable risk factors, such as muscle flexibility, range of motion, and individualized control of acute and chronic load. However, this possibility remains speculative and requires further research before practical recommendations can be made.

The higher incidence of muscle injuries among players with the XX genotype may be linked to a lack of α-actinin-3 in fast-twitch muscle fibers, as a result of the *ACTN3* rs1815739 polymorphism. This protein is crucial for withstanding high muscle stress [41]. Football, being an “explosive” sport involving high mechanical stress (jumps, sprints, direction changes [42], shows a lower proportion of players with the XX genotype (17% in our study across all participants) compared to other genotypes, with only 12% among professional players. These figures are consistent with previous research on elite footballers [43]. However, our sample also shows a notably high proportion of RR players (53% across all participants), which is unusual and likely explains the deviation from HWE in the overall sample. It is possible that this distribution is influenced by a selection bias within the club, where performance-related physical qualities, such as muscle strength and explosive power, often associated with the RR genotype, are favored during player recruitment. In addition, the high proportion of players of African ancestry in the U17 and U19 groups (Fig. 2) may also have contributed to the higher RR genotype frequency, as previous population studies have shown greater *ACTN3* R allele prevalence in African ancestry cohorts [54]. Indeed, football is a sport that demands repeated high-intensity actions, and players with the RR genotype may be better suited to these demands.

The deficiency in α-actinin-3 (XX compared to RR or RX genotypes) is generally associated with alterations in force transmission structures in the myofilaments at the Z-disc [44]. Given that α-actinin-3 plays a crucial role in anchoring actin filaments to the Z-disc, its deficiency could limit the muscle’s ability to endure the stresses associated with explosive sports actions, such as those in football [25]. Interestingly, it has also been found that, in addition to a higher injury incidence, XX players had lower match performance values than RR players, especially regarding actions performed at high-intensity such as sprints [36]. Overall, all the literature suggests that the *ACTN3* XX genotype impacts on muscle performance, and overall athletic ability, given its association with reduced fast-twitch muscle fiber function. This study demonstrates that the negative effect of the *ACTN3* XX genotype appears appreciable even in a sample younger population of players (U17 and U19). Although genetic testing has been proposed as a potentially convenient method for identifying young soccer players who may be more prone to injury, at present, the evidence remains insufficient to recommend its routine application. Instead, our findings highlight a possible avenue for future research, where genetic information could eventually be combined with other intrinsic and extrinsic risk factors to guide more individualized prevention strategies.

Regarding return-to-play time, our results indicate that the *ACTN3* R577X genotype significantly affected this variable. Median time to return after a muscle injury was around 13 days for RR players, compared to 16–18 days for RX and XX genotypes. These differences were statistically significant in the overall sample (*p* = 0.007) and particularly pronounced among U17 players (*p* = 0.004), while no significant differences were observed in professionals or U19 players (Table 3). The “wild type” R allele, often associated with greater strength and power [24], seems to facilitate a faster return to play after a muscle injury, unlike the “mutant” X allele. One possible reason for these findings might be that RR players sustained less severe muscle injuries during the study period compared to players carrying the X allele, which could explain their shorter return-to-play times, as previously found by other authors [45]. Previous studies on professional footballers have also shown that the *ACTN3* genotype influences recovery time, with XX players taking longer to return to play [31]. XX players averaged 36 days to return to training and competition, compared to approximately 17 to 20 days for RR and RX genotypes in a prior study [31]. Additionally, Clos et al. observed a trend towards a longer recovery time after moderate muscle injury in XX players compared to RR and RX genotypes (approximately 41, 22, and 33 days, respectively), although this trend did not reach statistical significance [35]. Our results tend to validate the existing literature for young soccer players.

Supporting this, there is evidence suggesting that the *ACTN3* genotype might influence susceptibility and progression of muscle injuries, with the X allele associated with an increased risk of inflammatory myopathies [46]. Elevated blood markers of muscle damage (creatine kinase, myoglobin) have been reported in athletes with the X allele compared to the RR genotype following marathons [29] or Ironman events [28] regardless of performance times. These findings further underscore the protective and reparative role of ACTN3 [47]. These results are debated in football. Some authors have found similar results in professional players [48], while others have reported opposing findings in U16 players [49]. The latter authors have hypothesized that in self-regulated activities like football, compared to standardized tasks, individuals with the *ACTN3*-RR/RX genotype are likely to exhibit superior performance in activities requiring strength and power. This could lead to increased levels of post-microtrauma muscle activity in RR/RX individuals. Although this is a valid theory, the vast majority of the studies suggest just it is true that RR-RX exhibit superior performance, but they are also less prone to non-contact muscle injury than XX players.

### Limitations and Strengths

The primary limitation of this study is the relatively small overall sample size (*n* = 76), which restricts the statistical power of the analyses. This limitation is particularly evident among professionals (*n* = 22) and within the XX genotype subgroup, which represented only 11% of professionals and 14% of U19 players. In addition, with a small cohort comes a limited number of non-contact muscle injuries available for analysis. This becomes particularly constraining when stratifying injuries by genotype, since within the category of “non-contact muscle injuries” there are multiple possible locations, each with distinct incidence patterns and underlying mechanisms. The reduced statistical power limits our ability to analyze injuries by specific location or type. Therefore, the results should be considered preliminary. Secondly, although exposure time to training and matches was accurately recorded for each player by the club’s routine monitoring system, we did not control player participation itself, as this was determined by the technical and medical staff of each team to which the player belonged. The soccer club had its own protocols for injury prevention, treatment, and return-to-play, which were the same for all teams, but they may differ from other soccer clubs. Further studies with larger and more diverse samples may be necessary to validate the findings regarding the association between the *ACTN3* R577X polymorphism, injury incidence, and return-to-play time in professional and developing footballers. Another limitation of this study is the diverse ethnic origin of the participants. It is well established that the distribution of the *ACTN3* R577X genotype differs across populations, for example between individuals of Caucasian and African ancestry. Given that our sample included players of different ethnic backgrounds, this factor could have influenced the observed genotype distribution and partly contributed to the higher proportion of RR players in our cohort compared with other studies. Last, this study was performed with a sample of only-male participants and the results should not be transferred to female football, especially because the negative impact of the *ACTN3* XX genotype seems lesser in women athletes [21]. A notable strength of our study is the high level of competition among participants and the diversity of ages, which underscores the need for future research with larger cohorts to confirm our results. Additionally, examining return-to-play times in relation to genotype and the type of muscle injury (e.g., grade 1, grade 2, etc.) would be valuable. Additionally, technical staff, especially those working with young developing players, should be aware of this data to provide special care to players with the XX genotype. Finally, it should be stressed that the *ACTN3* gene is only one of several genetic markers potentially associated with muscle injuries. Given the polygenic nature of these injuries, future research should analyze *ACTN3* genotypes in combination with other candidate genetic variants to better understand the genetic contribution to injury incidence and severity in soccer. One hypothesis for future research could be to study the impact of the ACTN3 genotype among those players who succeed in becoming professionals. In this highly competitive sport, a high injury incidence and limited availability can be a barrier for developing players seeking to secure a professional contract.

## Conclusions

In summary, young football players with the *ACTN3* XX genotype required significantly longer recovery times following non-contact muscle injuries compared with RR players, particularly among U17 footballers. Incidence rates of non-contact muscle injuries also tended to be higher in XX players than in RR and RX players, although these differences did not reach statistical significance in our sample, likely due to the limited statistical power. If these trends are confirmed by future research, they would suggest that α-actinin-3 deficiency associated with the *ACTN3* XX genotype may be a factor negatively impacting both injury susceptibility and return-to-play time in high-level young male football players. However, these results should be considered preliminary due to the small sample size. Further studies are needed to determine whether the XX genotype truly represents a “risk” condition that should be considered by the medical staff of professional football teams. As a potential future application, genetic testing may help identify players with an increased susceptibility to injury. This information could subsequently be used to inform the development of targeted injury-prevention strategies tailored to individual characteristics and genetic profiles, although current evidence does not yet support routine implementation.

## Data Availability

All data supporting the findings of this study are available upon a reasonable request to corresponding authors.

## Abbreviations

ACTN3: α-Actinin-3 gene
ANOVA: Analysis of variance
HWE: Hardy-Weinberg Equilibrium
IOC: International Olympic Committee
SNPs: Single nucleotide polymorphisms
χ^2^ test: Chi-square test
